# JWA reverses cisplatin resistance via the CK2—XRCC1 pathway in human gastric cancer cells

**DOI:** 10.1038/cddis.2014.517

**Published:** 2014-12-04

**Authors:** W Xu, Q Chen, Q Wang, Y Sun, S Wang, A Li, S Xu, O D Røe, M Wang, R Zhang, L Yang, J Zhou

**Affiliations:** 1 Department of Molecular Cell Biology and Toxicology, School of Public Health, Nanjing Medical University, 818 East Tianyuan Road, Nanjing 211166, People's Republic of China; 2 Laboratory of Cancer Biology, Biomedical Research Center, Sir Runrun Shaw Hospital, Zhejiang University, Hangzhou, People's Republic of China; 3 Department of Cell Biology, School of Basic Medical Sciences, Nanjing Medical University, 140 Hanzhong Road, Nanjing 210029, People's Republic of China; 4 Department of Cancer Research and Molecular Medicine, Norwegian University of Science and Technology, Trondheim 7491, Norway and Cancer Clinic, Levanger Hospital, Nord-Trøndelag Health Trust, Levanger 7600, Norway; 5 Department of Biomedical Sciences, School of Pharmacy, Texas Tech University Health Sciences Center, 1406 Coulter Street, Suite 1117, Amarillo, TX 79106, USA; 6 Department of Pharmaceutical Sciences, School of Pharmacy, Texas Tech University Health Sciences Center, 1406 Coulter Street, Suite 1116, Amarillo, TX 79106, USA; 7 Department of Molecular and Cellular Oncology, The University of Texas MD Anderson Cancer Center, Houston, TX 77030, USA; 8 Jiangsu Key Laboratory of Cancer Biomarkers, Prevention and Treatment, Cancer Center, 818 East Tianyuan Road, Nanjing 211166, People's Republic of China; 9 Key Laboratory of Modern Toxicology (NJMU), Ministry of Education, 818 East Tianyuan Road, Nanjing, 211166 People's Republic of China

## Abstract

Gastric cancer is the third most common malignancy in China, with a median 5-year survival of only 20%. Cisplatin has been used in first-line cancer treatment for several types of cancer including gastric cancer. However, patients are often primary resistant or develop acquired resistance resulting in relapse of the cancer and reduced survival. Recently, we demonstrated that the reduced expression of base excision repair protein XRCC1 and its upstream regulator JWA in gastric cancerous tissues correlated with a significant survival benefit of adjuvant first-line platinum-based chemotherapy as well as XRCC1 playing an important role in the DNA repair of cisplatin-resistant gastric cancer cells. In the present study, we demonstrated the role of JWA in cisplatin-induced DNA lesions and aquired cisplatin resistance in five cell-culture models: gastric epithelial cells GES-1, cisplatin-sensitive gastric cancer cell lines BGC823 and SGC7901, and the cisplatin-resistant gastric cancer cell lines BGC823/DDP and SGC7901/DDP. Our results indicated that JWA is required for DNA repair following cisplatin-induced double-strand breaks (DSBs) *via* XRCC1 in normal gastric epithelial cells. However, in gastric cancer cells, JWA enhanced cisplatin-induced cell death through regulation of DNA damage-induced apoptosis. The protein expression of JWA was significantly decreased in cisplatin-resistant cells and contributed to cisplatin resistance. Interestingly, as JWA upregulated XRCC1 expression in normal cells, JWA downregulated XRCC1 expression through promoting the degradation of XRCC1 in cisplatin-resistant gastric cancer cells. Furthermore, the negative regulation of JWA to XRCC1 was blocked due to the mutation of 518S/519T/523T residues of XRCC1, and indicating that the CK2 activated 518S/519T/523T phosphorylation is a key point in the regulation of JWA to XRCC1. In conclusion, we report for the first time that JWA regulated cisplatin-induced DNA damage and apoptosis through the CK2—P-XRCC1—XRCC1 pathway, indicating a putative drug target for reversing cisplatin resistance in gastric cancer.

Gastric cancer (GC) is the fifth most common human malignant tumor worldwide but third cause of cancer death.^1^ In 2012, there were 405 000 new GC cases diagnosed and 325 000 deaths in China.^1^ Current strategy for treatment of GC includes surgery with chemotherapy for potentially curable disease and chemotherapy only for advanced disease. Unfortunately, owing to intrinsic or acquired drug resistance, relapse and metastasis are common and result in high mortality of GC.^2^

Cisplatin is a widely used chemotherapeutic drug for treating various tumors including GC.^3^ Cisplatin triggers apoptosis by inducing DNA damage through crosslinking of the DNA.^4^ However, cancer cells often develop multiple mechanisms to overcome cisplatin-induced DNA damage and apoptosis, and lead to cisplatin resistance.^5, 6^ Two of the major systems activated are enhanced capability of DNA repair and anti-apoptosis signaling pathways.^7, 8^

XRCC1 is a key mediator of single-strand break DNA repair, and is involved in the process of cisplatin-induced DNA damage repair in various tumors.^9, 10, 11^ XRCC1 was found to identify and bind to DNA interstrand crosslinks induced by cisplatin.^12^ Moreover casein kinase 2 (CK2) phosphorylates XRCC1 and is required for its stability and efficient DNA repair.^13^ A selective small molecule inhibitor of CK2, CX-4945, was found to block the cisplatin-induced DNA repair response by decreasing the phosphorylation of XRCC1 at CK2-specific phosphorylation sites.^14^ This body of evidence indicates a critical role of XRCC1 and CK2 in cisplatin resistance.

The *JWA* gene, also known as ARL6ip5, was initially cloned from human tracheal bronchial epithelial cells after treatment with all-trans retinoic acid.^15^ Subsequent studies indicated that JWA is involved in the cellular responses to heat shock and chemical-mediated oxidative stresses.^16, 17^ Moreover, JWA functions as a base excision repair protein in oxidative-stress-induced DNA single-strand breaks in NIH-3T3 and HELF cells, as evidenced by the positive regulation of XRCC1 levels through MAPK signal pathway and protecting XRCC1 protein from ubiquitination and degradation by proteasome.^18, 19^ However, JWA is also a structurally novel microtubule-binding protein, which regulates cancer cell migration *via* MAPK cascades and mediates differentiation of leukemic cells.^20, 21, 22^ JWA significantly inhibits melanoma adhesion, invasion and metastasis *via* integrin aVb3 signaling.^23^ More recent data have shown that JWA is required for As_2_O_3_-induced apoptosis in HeLa and MCF-7 cells *via* reactive oxygen species and mitochondria-linked signal pathway or promoted p38 MAPK-linked tubulin polymerization.^24, 25^ These reports indicate that the JWA functions as a tumor suppressor for tumor initiation and development.

Recently, we reported the prognostic and predictive role of JWA and XRCC1 expression in GC. JWA and XRCC1 protein levels are significantly downregulated in GC lesions compared with adjacent noncancerous tissues, whereas platinum-based chemotherapy significantly improved overall survival in GC patients with low levels of tumoral JWA or XRCC1 expression.^26^ Subsequent studies indicated that overexpression of XRCC1 contributed to cisplatin resistance in GC cells and showed that XRCC1 protein was important for effective repair of cisplatin-induced DSBs in GC cells.^27^ However, the contribution of JWA to cisplatin resistance in GC and underlying mechanisms are not fully understood.

The objectives of the present study were to investigate the role of JWA in cisplatin resistance of GC cells and elucidate the underlying mechanisms of action. Our results demonstrated that JWA negatively regulated XRCC1 through the CK2—p-XRCC1 pathway in cisplatin-resistant GC cells. The JWA could be a valuable target for reversal of cisplatin resistance in human GC.

## Results

### JWA is required for repair of cisplatin-induced DSBs in normal gastric epithelial cells *via* upregulation of XRCC1

**Figure 1:**
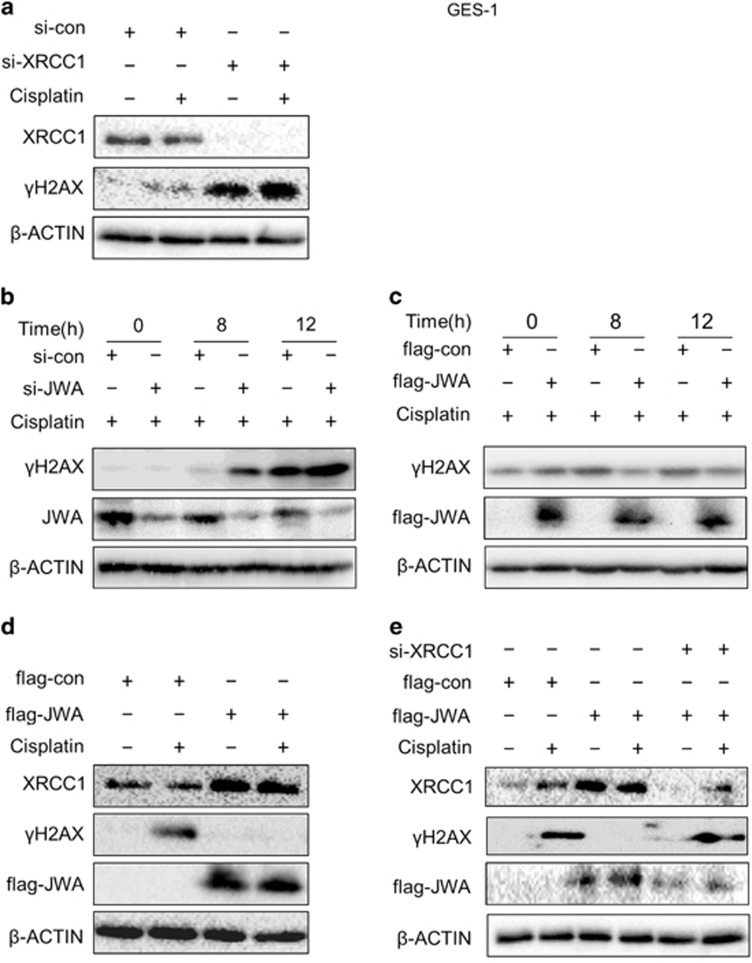
JWA is required for DNA repair following cisplatin-induced DSBs *via* XRCC1 in normal gastric epithelial cells. (**a**) The GES-1 cells were transfected with XRCC1 siRNA for 48 h followed by exposure to 3 *μ*g/ml of cisplatin for additional 24 h, and the western blotting was used to determine the expression of XRCC1 and *γ*H2AX. (**b**) The GES-1 cells were transfected with JWA siRNA for 48 h followed by exposure to 3 *μ*g/ml of cisplatin for additional 8 and 12 h, and western blotting was used to determine the expression of JWA and *γ*H2AX. (**c**) The GES-1 cells were transfected with flag-JWA for 48 h followed by exposure to 3 *μ*g/ml of cisplatin for additional 8 and 12 h, and western blotting was used to determine the expression of flag-JWA and *γ*H2AX. (**d**) The GES-1 cells were transfected with flag-JWA for 48 h followed by exposure to 3 *μ*g/ml of cisplatin for additional 12 h, and western blotting was used to determine the expression of flag-JWA, XRCC1 and *γ*H2AX. (**e**) The GES-1 cells were co-transfected with flag-JWA and XRCC1 siRNA for 48 h followed by exposure to 3 *μ*g/ml of cisplatin for additional 12 h, and western blotting was used to determine the expression of flag-JWA, XRCC1 and *γ*H2AX

Gastric epithelial GES-1 cells were used to study the role of JWA and XRCC1 in cisplatin-induced DSB repair where phosphorylated histone H2AX (*γ*H2AX) was employed as a sensitive surrogate marker of DSBs. First, knockdown of XRCC1 or JWA expression by small interfering RNA (siRNA) were confirmed by western blotting (Supplementary Figure 1a and b). Elevated *γ*H2AX levels were observed by inhibiting XRCC1 expression in GES-1 cells treated with 3 *μ*g/ml of cisplatin for 12 h (Figure 1a). Knockdown of JWA expression by transfection of JWA siRNA resulted in increased *γ*H2AX levels in the cells treated with 3 *μ*g/ml of cisplatin for 8 h and 12 h (Figure 1b). In contrast, the overexpression of JWA by transfection of flag-JWA plasmid resulted in decreased *γ*H2AX levels in the cisplatin-treated cells (Figure 1c). Overexpression of JWA lead to significantly increased XRCC1 levels and decreased *γ*H2AX levels (Figure 1d). Finally, *γ*H2AX levels increased by co-transfection of flag-JWA plasmid and XRCC1 siRNA compared with transfected flag-JWA alone (Figure 1e). These results suggested that JWA is required for the repair of cisplatin-induced DSBs through positive regulation of XRCC1 in human gastric epithelial cells.

### JWA enhances cisplatin-induced cell death through regulation of DNA damage-induced apoptosis in GC cells

**Figure 2:**
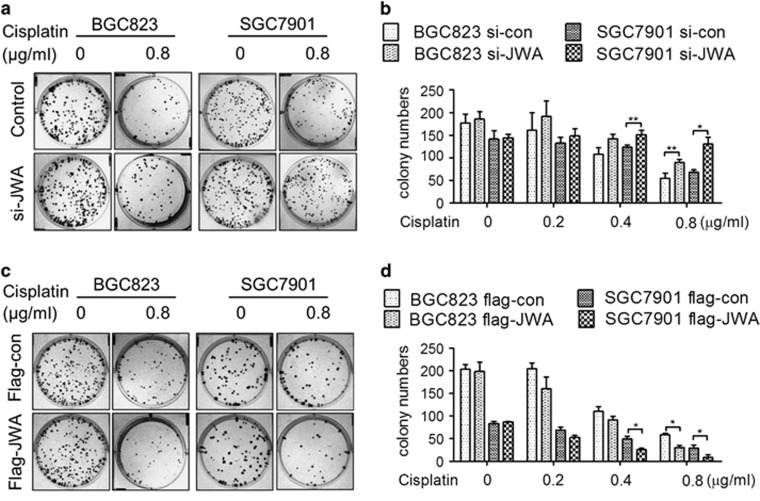
JWA enhances cisplatin-induced cell death in gastric cancer cells. (**a**) BGC823 and SGC7901 cells were transfected with JWA siRNA for 48 h and subjected to clonogenic survival assay 2 weeks after treatment with cisplatin for 2 h. (**b**) Quantify numbers of colony in BGC823 and SGC7901 cells transfected with JWA siRNA, each colony containing cells >50 were counted. (**c**) BGC823 and SGC7901 cells were transfected with flag-JWA for 48 h and subjected to clonogenic survival assay 2 weeks after treatment with cisplatin for 2 h. (**d**) Quantify numbers of colony in BGC823 and SGC7901 cells transfected with flag-JWA, each colony containing cells >50 were counted.*P<0.05, ^**^P<0.01

**Figure 3:**
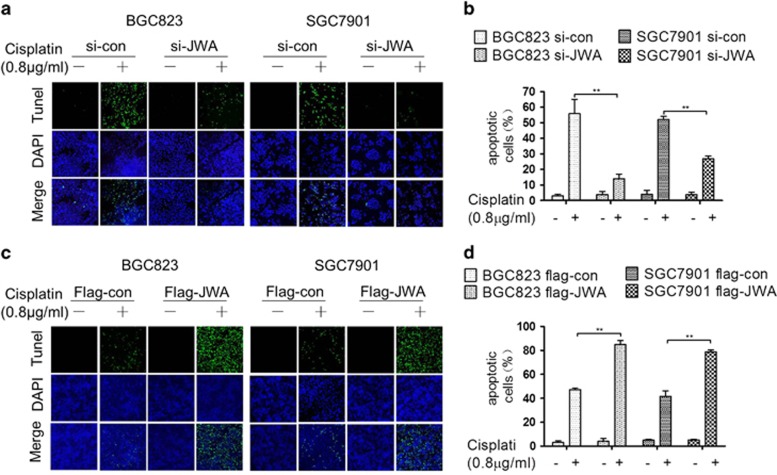
JWA enhances cisplatin-induced apoptosis in gastric cancer cells. (**a**) The BGC823 and SGC7901 cells were transfected with JWA siRNAfor 48 h and followed by exposure to 0.8 *μ*g/ml of cisplatin for 48 h, and the apoptotic rate was determined by the TUNEL assay ( × 100). (**b**) Quantification of TUNEL-positive BGC823 and SGC7901 cells transfected with JWA siRNA. (**c**) The BGC823 and SGC7901 cells transfected with flag-JWA for 48 h followed by exposure to 0.8 *μ*g/ml of cisplatin for 48 h, and the apoptotic rate was determined by the TUNEL assay ( × 100). (**d**) Quantification of TUNEL-positive BGC823 and SGC7901 cells transfected with flag-JWA. **P<0.01

It was previously reported that JWA is required for apoptosis induced by chemotherapeutics such as As_2_O_3_ and VP16 in cancer cells.^25, 28^ Here, two GC cell lines BGC823 and SGC7901 were used to investigate the role of JWA in apoptosis triggered by cisplatin-induced DSBs. Long-term clonogenic survival assay was employed to detect cisplatin-induced cell death. The colony numbers were significantly increased by treatment with 0.8 *μ*g/ml of cisplatin in JWA knockdown BGC823 or with 0.4 and 0.8 *μ*g/ml of cisplatin in JWA knockdown SGC7901 cells (Figures 2a and b). In contrast, the colony numbers were significantly decreased by treatment with 0.8 *μ*g/ml of cisplatin in JWA-overexpressing BGC823 cells or with 0.4 and 0.8 *μ*g/ml of cisplatin in JWA-overexpressing SGC7901 cells (Figures 2c and d). These results were also confirmed in JWA stable knockdown or overexpression cells (Supplementary Figure 2). Moreover, the TUNEL assay showed that the apoptotic rate was significantly decreased by treatment with 0.8 *μ*g/ml of cisplatin for 48 h in JWA knockdown BGC823 and SGC7901 cells by different JWA siRNA (Figures 3a and b,Supplementary Figure 3) . In contrast, the apoptotic rate was significantly increased by treatment with 0.8 *μ*g/ml of cisplatin for 48 h in JWA-overexpressing BGC823 and SGC7901 cells (Figures 3c and d).

**Figure 4:**
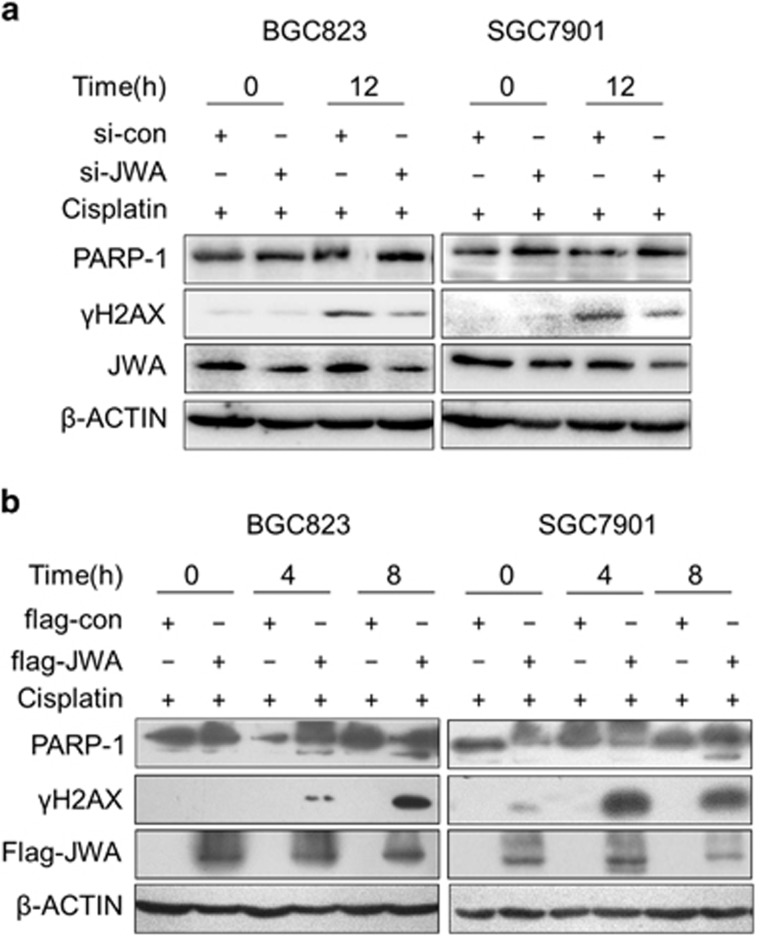
JWA enhances cisplatin-induced cell death through the regulation of DNA damage-induced apoptosis in gastric cancer cells. (**a**) The BGC823 and SGC7901 cells transfected with JWA siRNA for 48 h followed by exposure to 0.8 *μ*g/ml of cisplatin for additional 12 h, and western blotting was used to determine the expression of JWA, *γ*H2AX and PARP-1. (**b**) The BGC823 and SGC7901 cells were transfected with flag-JWA for 48 h and followed by exposure to 0.8 *μ*g/ml of cisplatin for additional 4 and 8 h, and western blotting was used to determine the expression of flag-JWA, *γ*H2AX and PARP-1

Then, the molecular base how JWA enhanced cisplatin-induced apoptosis was investigated. In JWA knockdown BGC823 and SGC7901 cells treated with 0.8 *μ*g/ml of cisplatin for 12 h, *γ*H2AX levels were significantly decreased, opposite to the findings in GES-1 (Figure 4a). In contrast, significantly increased *γ*H2AX levels and cleaved form of PARP1 (89 KD) levels were observed by treatment with 0.8 *μ*g/ml of cisplatin for 4 h or 8 h in JWA-overexpressing BGC823 and SGC7901 cells (Figure 4b).

### JWA downregulates XRCC1 in cisplatin-resistant GC cells

**Figure 5:**
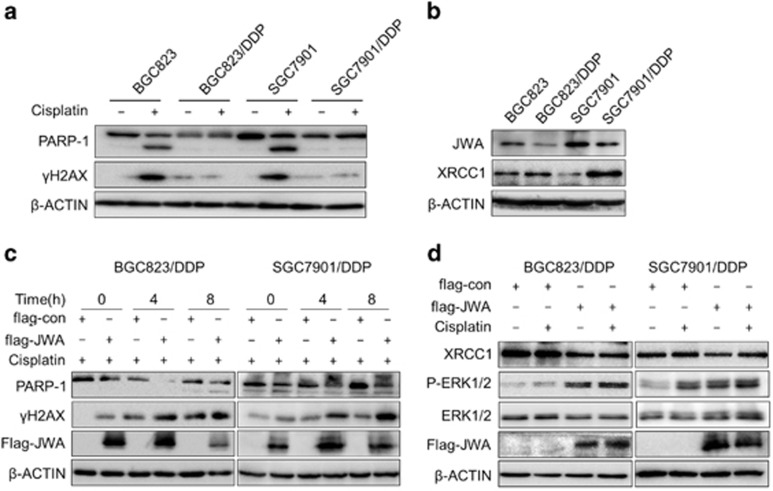
JWA negatively regulates XRCC1 in cisplatin-resistant gastric cancer cells. (**a**) The BGC823, BGC823/DDP, SGC7901 and SGC7901/DDP cells were exposure to 0.8 *μ*g/ml of cisplatin for 24 h, and western blotting was used to determine the expression of *γ*H2AX and PARP-1. (**b**) Western blotting was used to determine the expression of JWA and XRCC1 in BGC823, BGC823/DDP, SGC7901 and SGC7901/DDP cells. (**c**) The BGC823/DDP and SGC7901/DDP cells were transfected with flag-JWA for 48 h and followed by exposure to 10 *μ*g/ml of cisplatin for additional 24 h, and western blotting was used to determine the expression of flag-JWA, *γ*H2AX and PARP-1. (**d**) The BGC823/DDP and SGC7901/DDP cells were transfected with flag-JWA for 48 h and followed by exposure to 10 *μ*g/ml of cisplatin for additional 24 h, and western blotting was used to determine the expression of flag-JWA, ERK1/2, P-ERK12 and XRCC1

Recently, we have established two cisplatin-resistant GC cell lines BGC823/DDP cells and SGC7901/DDP. Previously, we noted that the enhanced capacity of DNA repair and anti-apoptosis in BGC823/DDP cells.^27^ Cisplatin resistance characteristics of these two cell lines was confirmed by comparison with their parental cell lines BGC823 cells and SGC7901 cells, respectively. After exposure to cisplatin, the cleaved form of PARP1 and *γ*H2AX were significantly elevated in BGC823 and SGC7901 than in BGC823/DDP and SGC7901/DDP cells measured by western blotting (Figure 5a). Then, we focused on the role of JWA and XRCC1 in these cisplatin-resistant cells. Consistent with our previous results, XRCC1 was overexpressed in cisplatin-resistant BGC823/DDP and SGC7901/DDP cells (Figure 5b), however, JWA was obviously decreased in these cells compared with their parental sensitive BGC823 and SGC7901 cells (Figure 5b). Accordingly, significantly increased *γ*H2AX levels and cleaved form of PARP1 (89 KD) levels were observed in JWA-overexpressed BGC823/DDP and SGC7901/DDP cells when treatment with 10 *μ*g/ml of cisplatin for 4 h or 8 h (Figure 5c). It seems that the expression patterns of JWA and XRCC1 were opposite in cisplatin-resistant GC cells compared with gastric epithelial cells. Our data showed that the levels of XRCC1 protein was downregulated when JWA was overexpressed in the BGC823/DDP and SGC7901/DDP cells (Figure 5d). These results indicated that JWA may enhance cisplatin-induced DNA damage and apoptosis by negatively regulating XRCC1 in cisplatin-resistant GC cells.

### Rescue JWA expression renders cisplatin-resistant GC cells more sensitive to cisplatin-induced apoptosis

**Figure 6:**
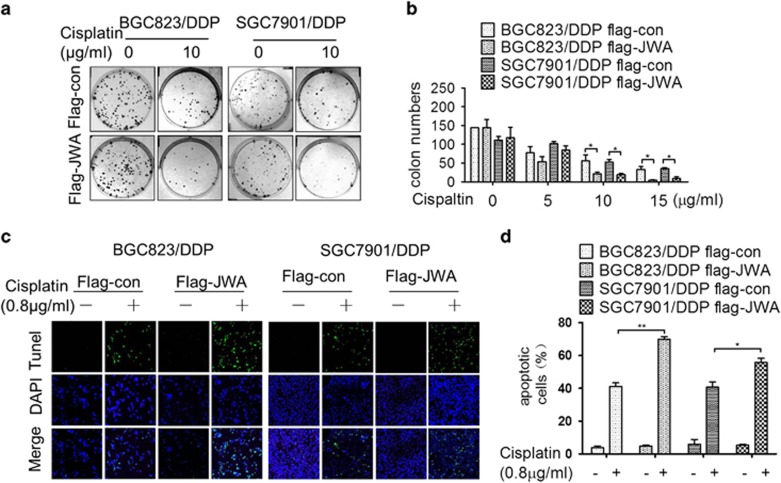
Rescued JWA expression in cisplatin-resistant gastric cancer cells causes more sensitive to cisplatin-induced apoptosis and cell death. (**a**) The BGC823/DDP and SGC7901/DDP cells were transfected with flag-JWA for 48 h and followed by exposure to 10 *μ*g/ml of cisplatin for 48 h, and the apoptotic rate was determined by the TUNEL assay ( × 100). (**b**) Quantification of TUNEL-positive BGC823/DDP and SGC7901/DDP cells transfected with flag-JWA. (**c**) The BGC823/DDP and SGC7901/DDP cells were transfected with flag-JWA for 48 h and subjected to clonogenic survival assay 3 weeks after treatment with cisplatin for 2 h. (**d**) Quantify numbers of colony in BGC823/DDP and SGC7901/DDP cells transfected with flag-JWA, each colony containing cells >50 were counted. *P<0.05, **P<0.01

In line with JWA-enhanced cisplatin-induced DNA damage, the long-term clonogenic survival assay showed that the colony numbers were significantly decreased in JWA-rescued BGC823/DDP and SGC7901/DDP cells by treatment with 10 or 15 *μ*g/ml of cisplatin (Figures 6a and b). The TUNEL assay also showed that the apoptotic rate was significantly increased by treatment with 10 *μ*g/ml of cisplatin for 48 h in the JWA-rescued cells (Figures 6c and d). These data demonstrated that JWA functions as a cisplatin resistance suppressor in GC cells.

### JWA downregulates XRCC1 expression *via* CK2—p-XRCC1 in cisplatin-resistant GC cells

**Figure 7:**
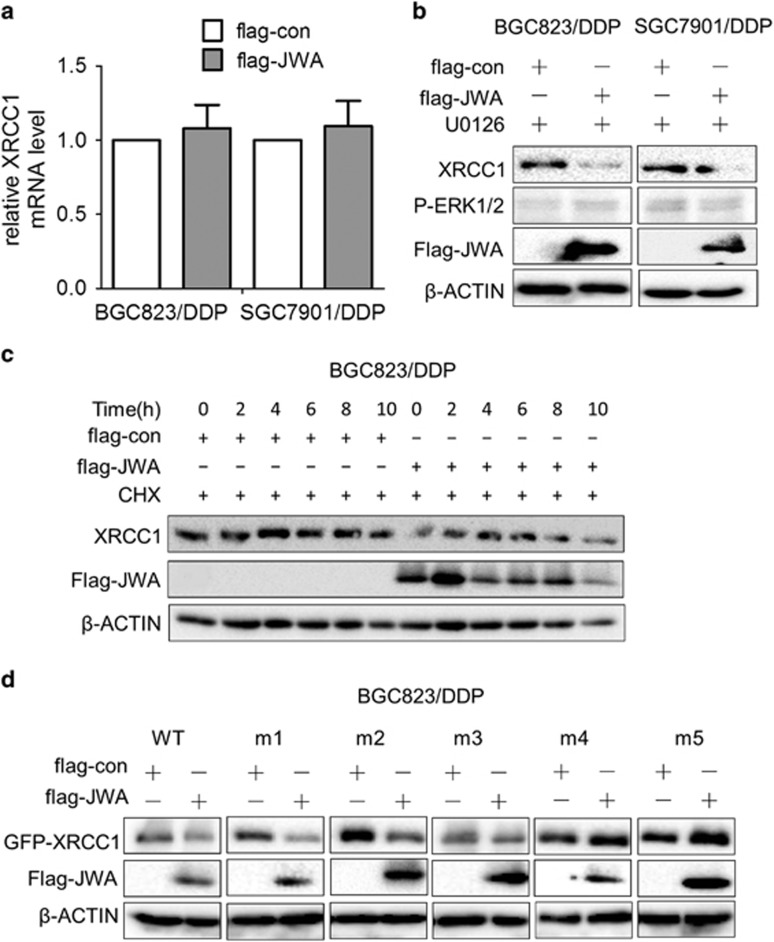
JWA negatively regulates XRCC1 expression by promoting the degradation of XRCC1 in cisplatin resistant gastric cancer cells. (**a**) The BGC823/DDP and SGC7901/DDP cells were transfected with flag-JWA for 48 h, and the XRCC1 mRNA levels in BGC823 and BGC823/DDP cells were analyzed by RT-PCR. (**b**) The BGC823/DDP and SGC7901/DDP cells were transfected with flag-JWA for 48 h followed by exposure to 20 *μ*g/ml of U0126 for additional 24 h, and western blotting was used to determine the expression of flag-JWA, P-ERK1/2 and XRCC1. (**c**) The protein stability of XRCC1 in BGC823/DDP cells transfected with flag-JWA for 48 h was assessed by western blotting analysis after treatment with 50 *μ*g/ml of cycloheximide at the indicated times. (**d**) The BGC823/DDP cells were co-transfected with flag-JWA and XRCC1-WT, XRCC1-m1, XRCC1-m2, XRCC1-m3, XRCC1-m4 and XRCC1-m5 for 48 h, and western blotting was used to determine the expression of flag-JWA and GFP-XRCC1

The mechanism behind JWA negative regulation of XRCC1 in cisplatin-resistant GC cells was examined. Our previous data showed that JWA upregulates the mRNA levels of XRCC1 through MEK-ERK1/2 signal pathway and protects XRCC1 protein from ubiquitination and degradation by proteasome in normal cells. Here, we first detected the mRNA levels and protein stability of XRCC1. Data showed that the mRNA levels of XRCC1 changed by overexpression of JWA in BGC823/DDP and SGC7901/DDP cells (Figure 7a), but the P-ERK1/2 levels were activated by the overexpression of JWA (Figure 5c). However, XRCC1 downregulation by JWA could not be blocked by U0126, a specific inhibitor of MEK-ERK1/2 pathway in both cells (Figure 7b). Then, transfection of flag-JWA or control plasmid into BGC823/DDP cells treated with cycloheximide for 0, 2, 4, 6, 8 and 10 h showed that the overexpression of JWA promoted the degradation of XRCC1 (Figure 7c). These results indicated a difference in JWA regulation of XRCC1 in normal gastric cells and cisplatin-resistant GC cells with positively regulated XRCC1 expression in normal cells, to promote the degradation of XRCC1 in cisplatin-resistant GC cells.

It was reported that phosphorylation of XRCC1 by a protein kinase, CK2, which is required for its efficacy on DNA repair where de-phoshorylated XRCC1 is ubiquitinylated by an E3 ubiquitin ligase and degraded by the proteasomal machinery.^13^ The S/T residues in amino acid sequence 403–538 of XRCC1 are known to be phosphorylated *in vitro* by CK2.^29, 30, 31^ To understand whether the degradation of XRCC1 promoted by JWA is *via* a CK2-related XRCC1 phosphorylation mechanism, the S/T residues in amino acids sequence 403–538 of XRCC1 were mutated from GFP-XRCC1 wild-type (XRCC1-WT) plasmid. The 461S was mutated to 461A labelled XRCC1-m1, and 475S was mutated to 475A labelled XRCC1-m2, 485S/488T was mutated to 485A/488A labelled XRCC1-m3, 518S/519T/523T was mutated to 518A/519A/523A labelled XRCC1-m4, and all these residues when mutated were labelled XRCC1-m5. Western blotting detected that the exogenous levels of GFP-XRCC1 protein were downregulated in BGC823/DDP cells co-transfected with flag-JWA and XRCC1-WT, XRCC1-m1, XRCC1-m2 and XRCC1-m3 (Figure 7d). However, the exogenous levels of GFP-XRCC1 protein were not affected in BGC823/DDP cells co-transfected with flag-JWA and XRCC1-m4 and XRCC1-m5 (Figure 7d). These results indicated that the mutation of XRCC1's 518S/519T/523T residues blocked the negative JWA regulation of XRCC1.

**Figure 8:**
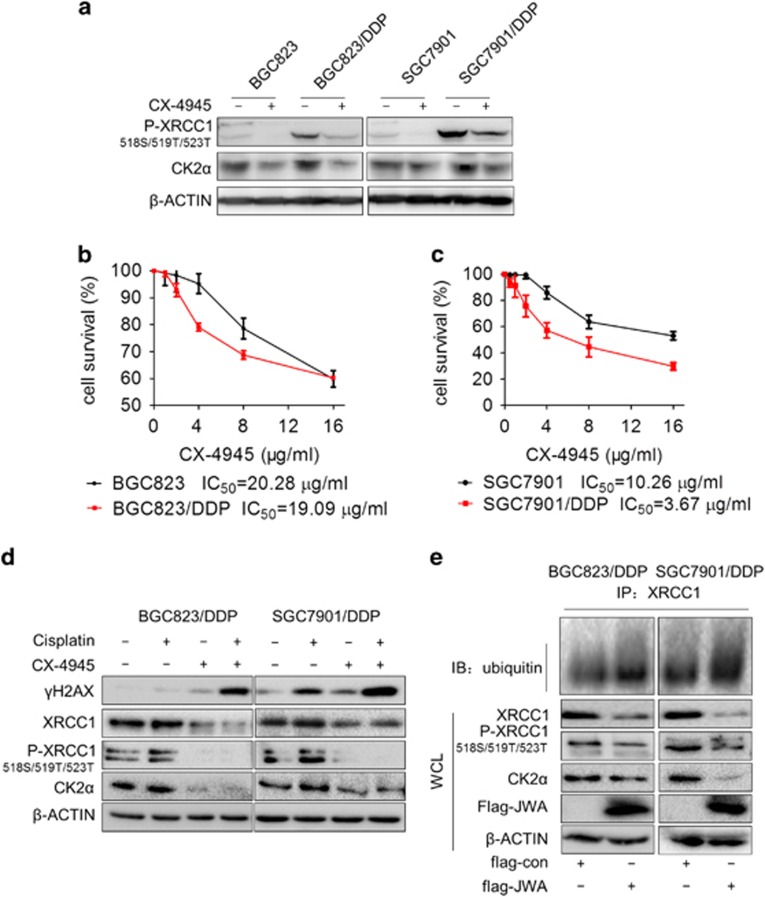
JWA negatively regulates XRCC1 expression *via* CK2—P-XRCC1 in cisplatin-resistant gastric cancer cells. (**a**) The BGC823, BGC823/DDP, SGC7901 and SGC7901/DDP cells were treated with 2 *μ*g/ml of CX-4945 for 24 h, and western blotting was used to determine the expression of CK2*α* and P-XRCC1(518S/519T/523T) in BGC823, BGC823/DDP, SGC7901 and SGC7901/DDP cells. (**b** and **c**) The cell viability was determined by exposure of BGC823, BGC823/DDP, SGC7901 and SGC7901/DDP cells to CX-4945 for 24 h. (**d**) Western blotting determined the levels of CK2*α*, P-XRCC1(518S/519T/523T), XRCC1 and *γ*H2AX in BGC823/DDP cells and SGC7901/DDP cells exposed to 2 *μ*g/ml of CX-4945 combined with 10 *μ*g/ml cisplatin for 24 h. (**e**) The BGC823/DDP and SGC7901/DDP cells were transfected with flag-JWA for 48 h, and western blotting was used to determine the expression of flag-JWA, CK2*α*, P-XRCC1(518S/519T/523T) and immunoprecipitation was employed to show the ubiquitination of XRCC1

Considerating that the 518S/519T/523T residues of XRCC1 are the primary CK2-phosphorylated domains, we investigated that whether the role of JWA was *via* the CK2-P-XRCC1 pathway. First, we found that the levels of CK2 catalytic subunit CK2*α* and P-XRCC1 (518S/519T/523T) were upregulated in BGC823/DDP and SGC7901/DDP cells compared with BGC823 and SGC7901 cells, respectively (Figure 8a). The CX-4945, a selective small molecule inhibitor of CK2 in clinical trial, blocks the cisplatin-induced DNA repair response by decreasing the phosphorylation of XRCC1 at CK2-specific phosphorylation sites.^14^ Here, we observed that the levels of CK2*α* and P-XRCC1 (518S/519T/523T) were inhibited by treatment with 2 *μ*g/ml of CX-4945 for 24 h in BGC823, BGC823/DDP, SGC7901 and SGC7901/DDP cells (Figure 8a). The viability was then determined by the CCK-8 assay in two cisplatin-sentitive and -resistant GC cells treated with CX-4945. Interestingly, the mean IC_50_ value of CX-4945 in BGC823/DDP cells (19.09 *μ*g/ml) was little lower than that of their parental BGC823 cells (20.28 *μ*g/ml) (Figure 8b). However, the mean IC_50_ value of CX-4945 in SGC7901/DDP cells (3.67 *μ*g/ml) was significantly lower than SGC7901 cells (10.26 *μ*g/ml) (Figure 8c). The sensitivity of CX-4945 correlated to the levels of CK2*α* protein in GC cells. When the BGC823/DDP cells and SGC7901/DDP cells were treated with a combination of 2 *μ*g/ml of CX-4945 and 10 *μ*g/ml of cisplatin for 24 h, the expression of Ck2*α*, p-XRCC1 (518S/519T/523T) and XRCC1 were inhibited although they could be activated by treatment with cisplatin alone. At the same time, *γ*H2AX levels were significantly enhanced in the combined group compared with the cells treated with either drug alone (Figure 8d). Finally, the expression of Ck2*α*, p-XRCC1(518S/519T/523T) and XRCC1 were downregulated and ubiquitination of XRCC1 was enhanced in JWA-overexpressed BGC823/DDP and SGC7901/DDP cells (Figure 8e). These results indicated that JWA regulated XRCC1 expression negatively *via* the CK2—p-XRCC1 signal pathway.

## Discussion

Through a series of mechanistic analyses of cisplatin-sensitive and -resistant cell lines, we report a novel pathway where cisplatin-induced DNA damage-apoptosis was regulated by JWA through the CK2—p-XRCC1—XRCC1 pathway.

As the anti-cancer activity of cisplatin was discovered by physicist B. Rosenberg in 1965,^32, 33, 34^ platinum-based therapy has become one of the most widely used chemotherapeutic treatments for cancers including lung, gastric, liver and ovarian cancer.^35^ In solid tumors, however, with the exception of testicular cancer, complete responses are rare and aquired resistance is more the rule than the exception.^36^

The cisplatin action mechanism is to damage DNA, primarily by formation of DSBs followed by intra- and inter-strand crosslinks, which consequently distort the DNA helix, inhibit DNA replication and drive cells into apoptosis.^37, 38^ Overexpression of DNA repair-associated proteins recruited to repair the damaged DNA and impaired cisplatin-induced apoptosis play crucial roles in the development of the cisplatin resistance.^39, 40, 41, 42^ We established two cisplatin-resistant cell lines BGC823/DDP and SGC7901/DDP to investigate the mechanism of cisplatin resistance in GC. The cisplatin-resistant cell lines BGC823/DDP have been shown to exhibit increased DNA repair and anti-apoptosis capacity.^27^ Here, we confirmed that both cisplatin-resistant cell lines have increased DNA repair and anti-apoptosis capacity.

Recent publications, including our reports, demonstrated an important role of XRCC1 in repair of cisplatin-induced DNA damage and apoptosis in cancer cells.^9, 11, 27, 43^ The JWA, an upstream regulator of XRCC1, was demonstrated to be involved in cellular responses to environmental stress including oxidative stress by upregulating the expression of XRCC1^18, 19^ and demonstrated the prognostic and predictive role of XRCC1 and JWA protein expressions in GC tissue.^26^

The current results revealed several novel connection and roles of JWA in cisplatin resistance. JWA was required for DNA repair following cisplatin-induced DSBs *via* XRCC1 in normal gastric epithelial cells, but JWA enhanced cisplatin-induced cell death through the regulation of DNA damage-induced apoptosis in GC cells. This could indicate that JWA promoted cisplatin-induced cell death in cancer cells and protecting the normal cell from the side-effects. Surprisingly, although JWA and XRCC1 function as DNA repair proteins had synergistic effect in normal gastric epithelial cells, opposite effects were shown in cisplatin resistant gastric cancer cells. XRCC1 was overexpressed in normal cells and in cisplatin-resistant cancer cells when challenged with cisplatin. Previous studies indicated a tumor suppressor role of JWA as it was downregulated during tumor development, for example, knockdown of JWA expression promoted tumor metastasis by integrin aVb3 signaling in melanoma.^23^ Inhibiting expression of JWA promoted angiogenesis in melanoma and GC.^44, 45^ Knockdown expression of JWA lead to increased survival of HeLa and MCF-7 cells by treatment with As_2_O_3_.^25^

Drug resistance can be innate or acquired. Our study model is of acquired resistance as the parental cells were sensitive. The dynamic state of JWA and XRCC1 expression related to chemotherapeutics needs further study.

CK2 appears to play a central role in the regulation of gene expression and protein synthesis/degradation by acting at different levels on the complex apoptotic machinery.^46^ More than 300 substrates of CK2 have been discovered and is involved in many diverse pathways.^47, 48^ Cell death pathways triggered by DNA damage can be regulated by CK2^49, 50^ involving the phosphorylation of XRCC1.^13, 51^ In the present study, we observed upregulated levels of CK2 catalytic subunit CK2*α* and P-XRCC1 (518S/519T/523T) in cisplatin-resistant cells, which were inhibited by CK2 inhibitor CX-4945. We here provided evidence that CX-4945 effectively inhibited the XRCC1 expression through inhibiting CK2-activated p-XRCC1 (518S/519T/523T) phosphorylation. The CX-4945 selectivity killed cisplatin-resistant cells according to the levels of CK2*α*. Moreover, a synergistic effect of CX-4945 combined with cisplatin triggered DNA damage in cisplatin-resistant cells. These data indicated that CK2 is a useful target for reversing cisplatin resistance in gastric caner cells.

We know that JWA positively regulated the mRNA levels of XRCC1 through MEK-ERK1/2 signal pathway and protects XRCC1 protein from ubiquitination and degradation by proteasome in normal cells. However, JWA downregulated XRCC1 levels through promoting the degradation of XRCC1 in cisplatin-resistant GC cells. In consideration of the CK2-regulated XRCC1 phosphorylation for its stability, we presumed JWA may negatively regulate XRCC1 expression through CK2-regulated XRCC1 phosphorylation machinery. Our data showed that the mutation of 518S/519T/523T residues blocked the negative regulation of JWA on XRCC1, indicating the critical role of 518S/519T/523T phosphorylation in negative regulation of XRCC1 by JWA. Overexpression of JWA lead to the inhibition of CK2—p-XRCC1—XRCC1 pathway. The mechanism discovered in the present study is novel.

**Figure 9:**
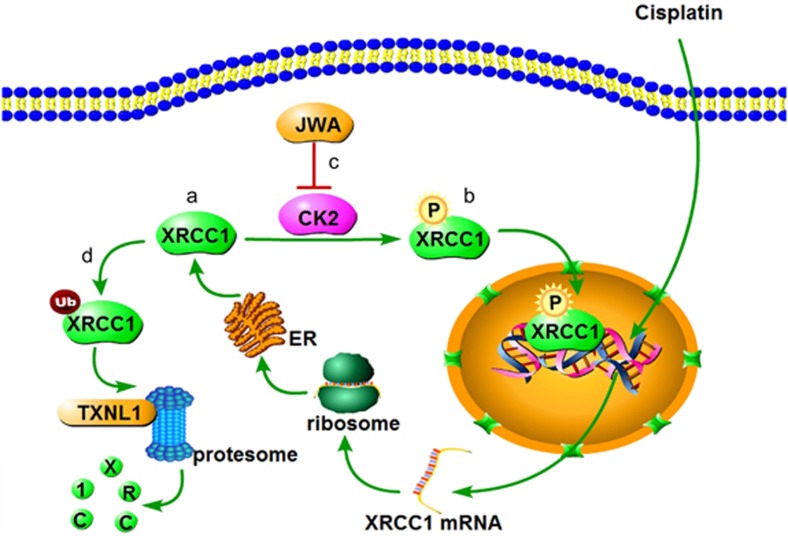
Diagram illustrates the proposed molecular signaling of JWA that reverses cisplatin resistance *via* the CK2—XRCC1 pathway in human gastric cancer. (**a**) XRCC1 expression was upregulated by cisplatin. (**b**) Overexpressed XRCC1 was phosphorylated by CK2. (**c**) JWA inhibited the expression of CK2. (**d**) Ubiquitination of XRCC1 was enhanced by the inhibition of CK2

Taken together, we report for the first time that JWA regulated cisplatin-induced DNA damage-apoptosis through the CK2—p-XRCC1—XRCC1 pathway (Figure 9). We therefore speculate that JWA is a potentially useful drug target and resistance biomarker for adjuvant chemotherapy with platinum-based regimen in GC. Further evaluation of these biomarkers in prospective clinical studies is warranted.

## Materials and Methods

### Cell lines and culture

Human gastric epithelial cells GES-1, GC cell lines BGC823 and SGC7901 were purchased from the Type Culture Collection of the Chinese Academy of Sciences (Shanghai, China). All the cells were cultured in RPMI 1640 medium supplemented with 10% of fetal bovine serum, 100 U/ml of penicillin and 100 *μ*g/ml of streptomycin (Life Technologies/Gibco, Grand Island, NY, USA). The cells were grown at 37 °C in a humidified incubator with 5% CO_2_. Cisplatin was obtained from Sigma-Aldrich (St. Louis, MO, USA). The cisplatin-resistant BGC823/DDP cells were developed from the parental BGC823 cells that were subjected to persistent gradient exposure to cisplatin for about 12 months, through increasing cisplatin concentration from 0.05 *μ*g/ml until the cells acquired resistance to 1 *μ*g/ml.^27^ The cisplatin-resistant SGC7901/DDP cells were obtained by the same way. Prior to each experiment, BGC823/DDP and SGC7901/DDP cells were cultured in drug-free RPMI 1640 medium for 2 weeks.

### Plasmids and transfection

The Flag-vector and Flag-JWA plasmid were kindly provided by Professor Gang Li (University of British Columbia, Canada) has been described previously.^25^ The GFP-XRCC1 plasmid was constructed from the RFP-XRCC1 plasmid that has been described previously.^19^ siRNA specific for XRCC1 ((si-XRCC1 5′-GGUCCUUCUAUAUCUGUAAdTdT-3′) and (si-XRCC1′ 5′- CGAUGGAUCUACAGUUGCA-3′)) and siRNA for JWA ((si-JWA 5′-CGAGCTATTTCCTTATCTC-3′) and (si-JWA′ 5′-AAGACCAUGACUCCUCCAAACAUGG-3′)) were synthesized (Ribobio, Guangzhou, China). The JWA siRNA expression cassettes were subcloned into the vector pGPU6/GFP/Neo to produce JWA shRNA. The plasmid DNA or siRNA was transfected into cells with Lipofectamine 2000 (Life Technologies, Grand Island, NY, USA) according to the manufacturer's instructions. Cells stably knockdown or overexpress JWA were generated following standard protocols by selection with 600 *μ*g/ml neomycin (G418) (Life Technologies/Gibco).

### Cytotoxicity assay

One day before treatment, BGC823, SGC7901, BGC823/DDP and SGC7901/DDP cells were plated at a density of 5000 cells per well in 96-well plates. The cells were treated with various concentrations of CX-4945 (Selleck, Shanghai, China). After 24 h, the cell viability was determined using Cell Counting Kit-8 (CCK-8) according to the manufacturer's instructions (Dojindo, Kumamoto, Japan). The cell variable curves were plotted and the IC_50_ values were evaluated through non-linear regression analysis by Graph Pad Prism software (La Jolla, CA, USA). The cell survival rates were expressed as mean±S.D. from at least three independent experiments.

### Clonogenic assay

After 48 h, the transfected BGC823, SGC7901, BGC823/DDP and SGC7901/DDP cells were treated with cisplatin at indicated concentrations for 2 h; and the cells were then trypsinized, seeded in six-well plates (300 cells per well) and cultured for further 2 weeks to BGC823 and SGC7901 cells or 3 weeks to BGC823/DDP and SGC7901/DDP cells, respectively. For scoring colonies, the cells were fixed in 1 ml of methanol for 15 min and stained with Giemsa for 10 min. The numbers of cloning were expressed as mean±S.D. from at least three independent experiments.

### Apoptosis assay

Apoptosis was determined using the TUNEL apoptotic cell detection kit (Roche, Basel, Switzerland), according to the manufacturer's instructions. The percentages of apoptotic cells were counted from at least 1000 cells.The confocal images of cells were sequentially acquired with Zeiss AIM software on a Zeiss LSM 700 confocal microscope system (Carl Zeiss Jena, Oberkochen, Germany).

### Western blotting

Western blot analyses were performed as previously described.^24^ The antibodies used were as follows: monoclonal anti-JWA (1 : 500, contract produced by AbMax, Beijing, China); monoclonal anti-*β*-actin, flag (1 : 2000, Beyotime, Haimen, Jiangsu, China); monoclonal anti-XRCC1, *γ*H2AX (1 : 2000, Epitomics, Burlingame, CA, USA); monoclonal anti-P-XRCC1(518S/519T/523T) (Bethyl Laboratories, Montgomery, TX, USA); monoclonal anti-CK2*α* (Santa Cruz, Dallas, TX, USA); monoclonal anti-PARP-1, P-ERK1/2, ERk1/2 (1 : 1000, Cell Signaling Technology, Danvers, MA, USA); cycloheximide and were purchased from Sigma-Aldrich.

### Immunoprecipitation

Immunoprecipitations were performed as previously described.^19^ Briefly, the cells were harvested and lyzed in cold lysis buffer (50 mMTris (pH 7.4), 150 mMNaCl, 1 mM EDTA, 0.5% (v/v) NP-40, 10% (v/v) glycerol,1 mM PMSF). The cell extracts were centrifuged at 12 000 × *g* at 4 °C for 15 min, and the supernatant was then incubated with protein A/G agarose beads (Santa Cruz) as a pretreatment. Precleared lysates were then incubated with anti-XRCC1 antibody or control IgG for 1 h, and then incubated overnight with protein A/G agarose beads. The beads were collected by centrifugation, washed three times with the lysis buffer and resuspended in 1 × SDS loading buffer. The immunoprecipitates were eluted from the beads by incubation at 95 °C for 5 min. The eluted proteins were separated by SDS-PAGE and western blotting was subsequently performed with ub antibodies.

### RNA extraction and reverse transcription-PCR

Total RNA was extracted from the cells using the Trizol reagent (Gibco, Gaithersburg, MD, USA) and quantified by UV spectrometry. Approximately 1 *μ*g of RNA was used for the reverse transcription reaction with OligodT (18T) (Life Technologies). The cDNA was amplified with the following primers: 5′- CATGTGGGCCATGAGGTCCACCAC -3′ (forward) and 5′- GGGAAGCTCACTGGCATGGCCTTCC -3′ (reverse) for GAPDH; 5′- ACTGCTGGAACCTGGCCCTGC -3′ (forward) and 5′- GCAAACCCCGAGGAGAAGGCA -3′ (reverse) for XRCC1. The following thermal cycling conditions were used: denaturation at 94 °C for 5 min followed by 36 cycles of denaturation at 94 °C for 35 s, annealing at 56 °C for 30 s and extension at 72 °C for 35 s.

### Statistical analysis

Data are expressed as the mean±S.D. The statistical significance of the differences between the cell lines was determined by the parametric unpaired Student's *t* test. Differences were considered significant when *P*<0.05.

## Abbreviations

DDP: cis-diammine dichloridoplatinum(II)
CK2: casein kinase 2
DSB: double-strand break
ERK: extracellular signal regulated kinase
GC: gastric cancer
JWA: ADP-ribosylation-like factor 6 interacting protein 5
MEK: mitogen-activated protein kinase
PARP1: poly(ADP-ribose) polymerase 1
TXNL1: thioredoxin-like protein 1
TUNEL: TdT-mediated dUTP Nick-End Labeling
Ub: Ubiquitin
XRCC1: X-ray repair cross complementing group1
*γ*H2AX: phosphorylated histone H2AX
