# Supplementary material for: JWA reverses cisplatin resistance via the CK2—XRCC1 pathway in human gastric cancer cells
Source: Cell Death Dis. 2014 Dec 4;5(12):e1551–. doi: 10.1038/cddis.2014.517 (PMC4649833; doi:10.1038/cddis.2014.517)

Supplementary figure 1

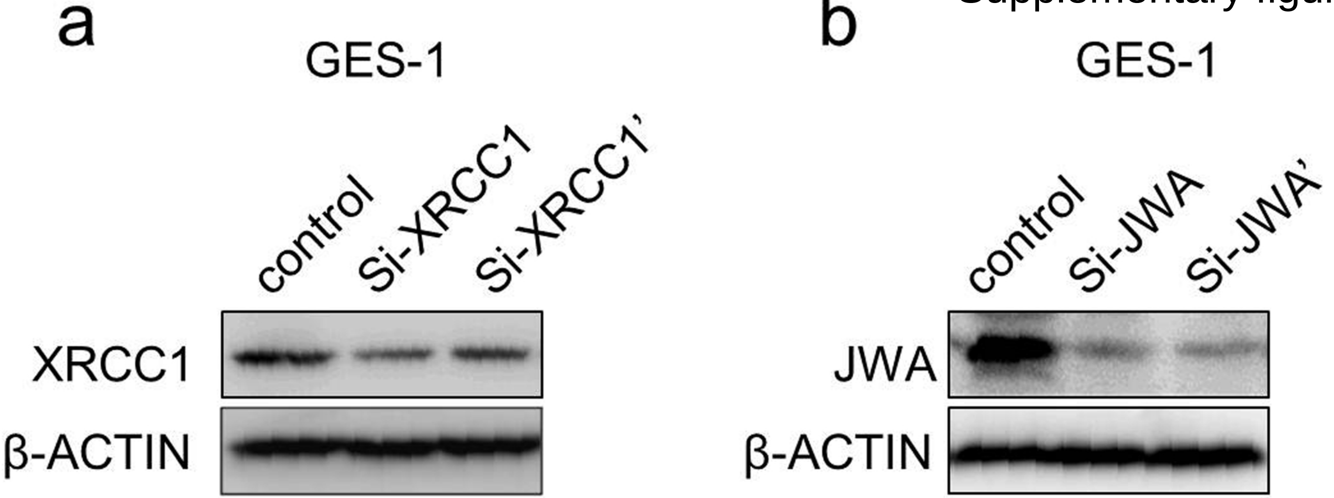

Supplement: Supplementary Figure 1 [file cddis2014517x1.pdf]
