# Supplementary figures and images for: JWA reverses cisplatin resistance via the CK2—XRCC1 pathway in human gastric cancer cells
Source: Cell Death Dis. 2014 Dec 4;5(12):e1551–. doi: 10.1038/cddis.2014.517 (PMC4649833; doi:10.1038/cddis.2014.517)

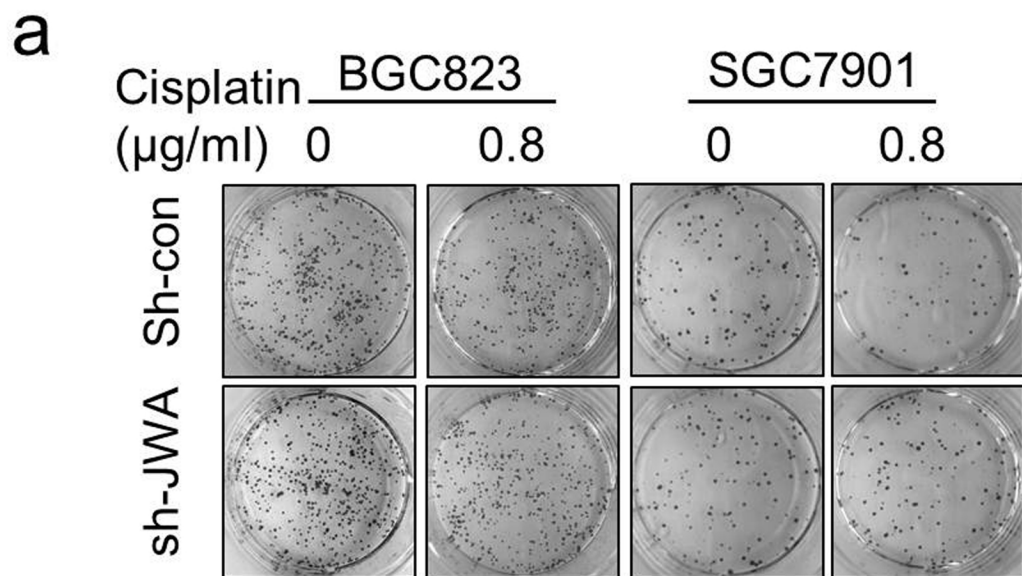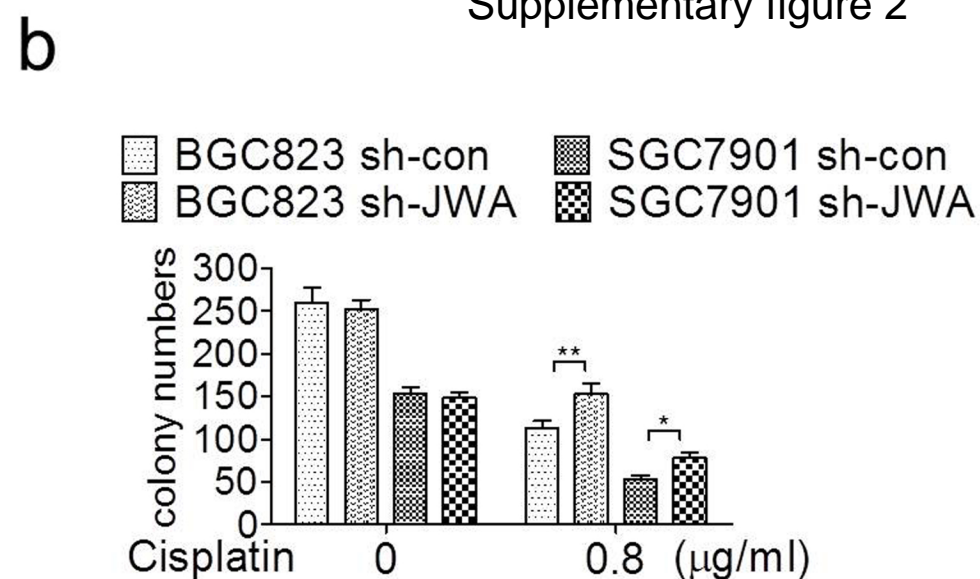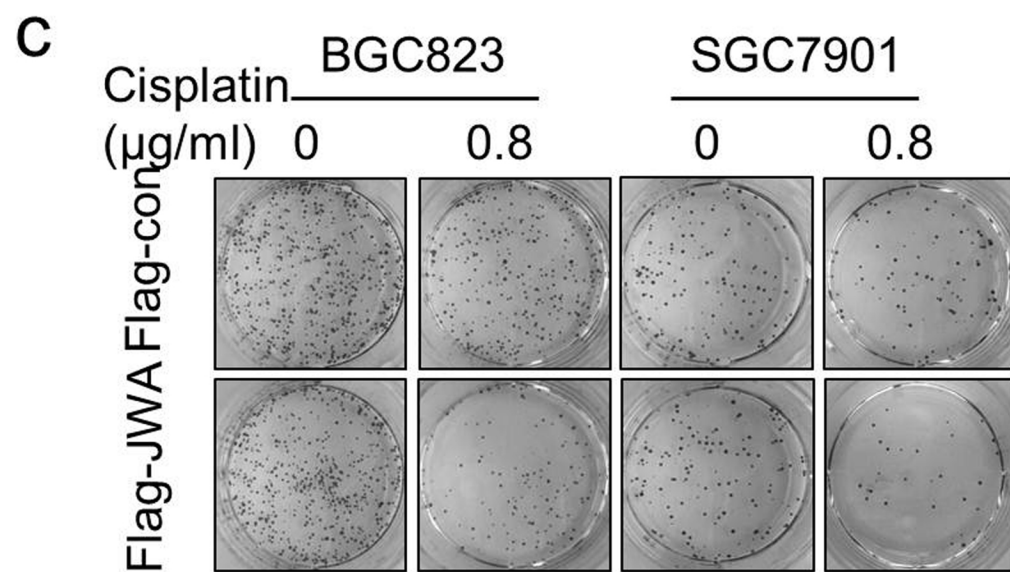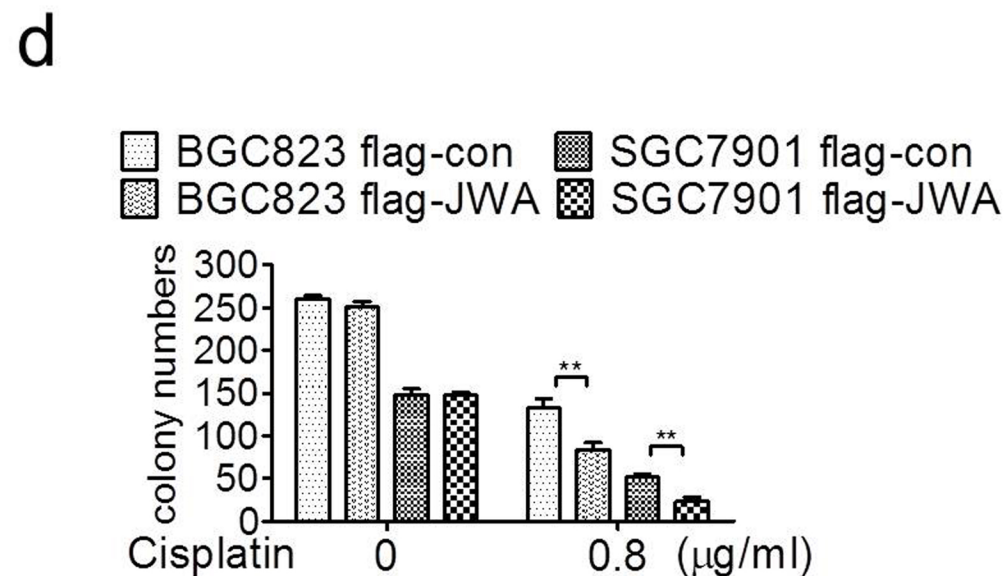

Supplement: Supplementary Figure 2 [file cddis2014517x2.pdf]

a

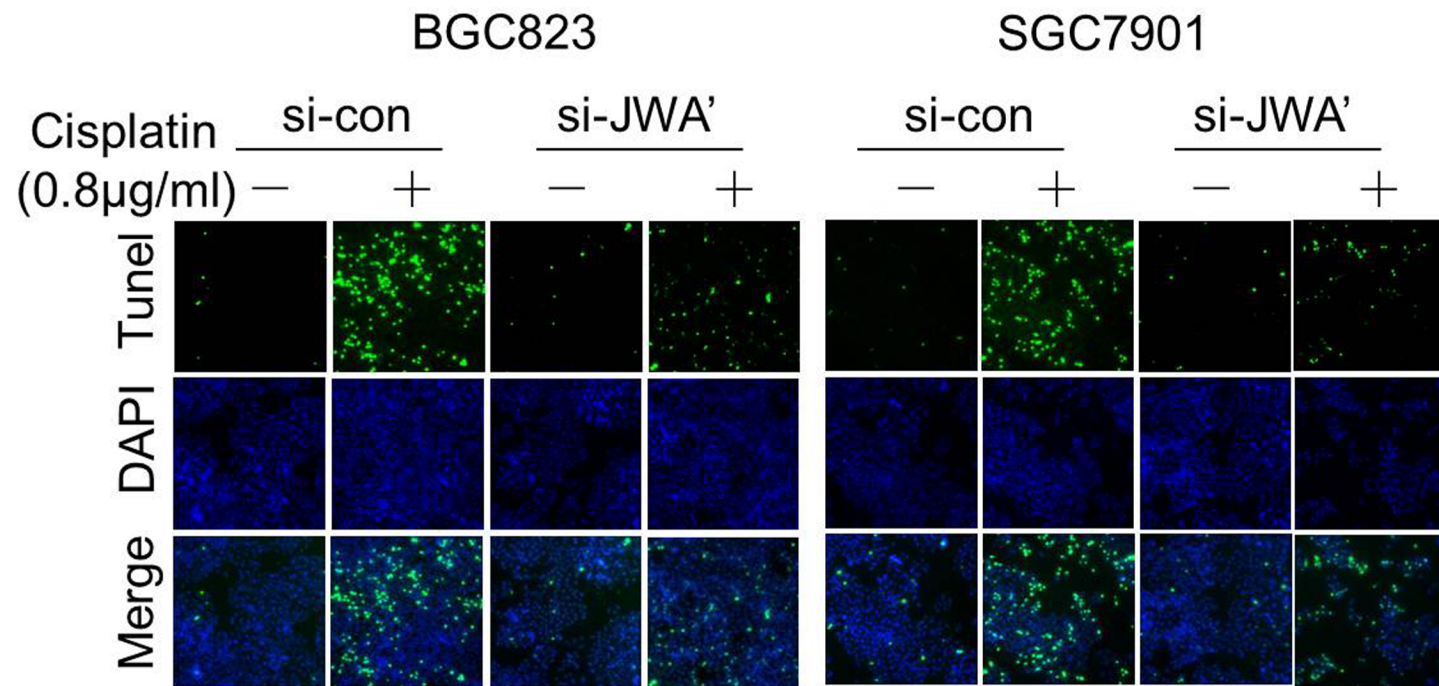

b

Supplementary figure 3

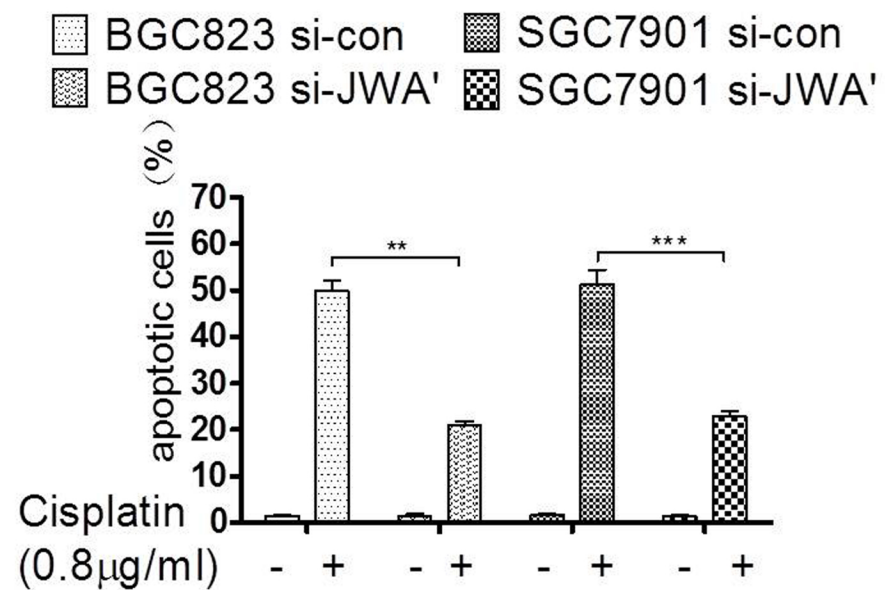

Supplement: Supplementary Figure 3 [file cddis2014517x3.pdf]
