# Supplementary material for: JWA reverses cisplatin resistance via the CK2—XRCC1 pathway in human gastric cancer cells
Source: Cell Death Dis. 2014 Dec 4;5(12):e1551–. doi: 10.1038/cddis.2014.517 (PMC4649833; doi:10.1038/cddis.2014.517)
Supplement: Supplementary Figure Legends [file cddis2014517x4.doc]

**Supplementary Figure Legends**

**Supplementary Figure 1. Knockdown of XRCC1 or JWA expression by siRNA，respectively.** (a) The GES-1 cells were transfected with different XRCC1 siRNA (si-XRCC1, si-XRCC1’) for 48h, and the Western blotting was used to determine the expression of XRCC1. (b) The GES-1 cells were transfected with different JWA siRNA (si-JWA, si-JWA’) for 48h, and the Western blotting was used to determine the expression of JWA.

**Supplementary Figure 2. JWA enhances cisplatin induced cell death in gastric cancer cells.** (a) BGC823 and SGC7901 cells were stablely transfected with JWA shRNA then subjected to clonogenic survival assay 2 weeks after treatment with cisplatin for 2 h. (b) Quantify numbers of colony in BGC823 and SGC7901 cells stablely transfected with JWA shRNA, each colony containing cells>50 were counted. (c) BGC823 and SGC7901 cells were stablely transfected with flag-JWA and subjected to clonogenic survival assay 2 weeks after treatment with cisplatin for 2 h. (d) Quantify numbers of colony in BGC823 and SGC7901 cells stablely transfected with flag-JWA, each colony containing cells >50 were counted.^*^*P*<0.05, ^**^*P*<0.01.

**Supplementary Figure 3. JWA enhances cisplatin induced apoptosis in gastric cancer cells.** (a) The BGC823 and SGC7901 cells were transfected with JWA siRNA (si-JWA’) for 48 h and followed by exposure to 0.8μg/ml of cisplatin for 48 h, and the apoptotic rate was determined by the TUNEL assay (×100). (b) Quantification of TUNEL positive BGC823 and SGC7901 cells transfected with JWA siRNA (si-JWA’). ^**^*P*<0.01, ^***^*P*<0.001.
